# Comparing Disparate Application of Physical Restraints on Patients Seeking Emergency Mental Health Care at Two San Francisco Emergency Departments

**DOI:** 10.1177/24731242251394114

**Published:** 2026-03-04

**Authors:** Drake Johnson, Lizbeth Alvarez, Anneka Hooft, Kenshata Watkins, Monique LeSarre, Aude Bouganon, Vanessa Jackson, Toni Hines, Lillian Agyei, Shanell Williams, Rosny Daniel, Alison Hwong, Will Martinez, Marilyn D. Thomas, Vidya Eswaran

**Affiliations:** 1 Department of Emergency Medicine, University of California San Francisco, San Francisco, California, USA.; 2 University of California Davis School of Medicine, Davis, California, USA.; 3 Department of Pediatrics, University of California San Francisco, San Francisco, California, USA.; 4 YWCA Berkley/Oakland, Berkeley, California, USA.; 5 School of Medicine, University of California San Francisco, San Francisco, California, USA.; 6 Rafiki Coalition for Health and Wellness, San Francisco, California, USA.; 7 Stanford Department of Psychiatry and Behavioral Sciences and VA Palo Alto, Palo University of California San Francisco, San Francisco, California, USA.; 8 UCSF Department of Psychiatry and Behavioral Sciences, San Francisco, California, USA.; 9 Department of Epidemiology and Biostatistics, University of California San Francisco, San Francisco, California, USA.; 10 Department of Emergency Medicine, Washington University St. Louis, St. Louis, Missouri, USA.

**Keywords:** psychiatric emergency, mental health, emergency department, physical restraint

## Abstract

**Introduction:**

Black patients are more likely to receive physical restraints in both emergency department (ED) and inpatient settings, yet the mechanisms behind these disparities remain unclear. This study aims to assess whether hospital type—academic tertiary care center versus county-funded trauma hospital—drives the association between race and restraint use.

**Methods:**

We used a community-based participatory research approach to analyze electronic medical record data from patients seen for a mental health emergency in 2019 and 2020 at two San Francisco hospitals, University of California, San Francisco Parnassus Hospital (UCSF) and Zuckerberg San Francisco General Hospital (ZSFG). Descriptive statistics and chi-square tests assessed patient characteristics by restraint, followed by multivariable logistic regression to estimate associations between race and restraint, stratified by hospital.

**Results:**

We identified 6,631 unique encounters, with 1,051 (15.8%) involving physical restraints. At ZSFG ED, 19.2% of patients were restrained, and 12.6% at UCSF ED. In combined adjusted hospital analysis, Black patients had higher odds of restraint (adjusted odds ratio [aOR] 1.54, 95% confidence interval [CI] 1.16–2.05). Stratified analysis showed significantly higher odds of restraint for Black patients at UCSF ED (aOR 1.77, 95% CI 1.12–2.79), while ZSFG ED showed marginally higher odds of restraint for Black patients (aOR 1.44, 95% CI 0.99–2.09).

**Discussion:**

Our findings suggest that hospital type may not significantly influence disparate restraint use in EDs. Further research should explore other potential contributors, including prehospital interactions with emergency services.

**Health Equity Implications:**

These results highlight the need for interventions that address the broader impact of structural racism on patients in mental health crises.

## Introduction

Black patients in the USA face inequities driven by systemic, institutional, cultural, interpersonal, and internalized racism. These disparities are pronounced in mental health care and may be exacerbated when receiving emergency services. Emergency Departments (EDs) serve as critical access points for patients with mental health concerns, with the proportion of ED visits related to mental health rising from 7% in 2007 to 11% in 2016.^1^

Compared with White Americans, Black Americans are more likely to be diagnosed with psychotic disorders,^2^ perhaps due to inappropriate overdiagnosis,^2^ yet access mental health care far less frequently.^3^ Despite higher prevalence of substance use and psychotic disorders,^4^Black Americans are less likely to receive appropriate treatment.^5^ Disparities in mental health care extend to coercive interventions, with Black patients experiencing physical restraints^6^ and/or court-ordered involuntary holds^7^ at higher rates than White patients.

San Francisco (SF) is a city that faces significant racial and socioeconomic disparities in health care access, shaped by historical redlining, structural inequities,^8^ and rising homelessness,^9^ further compounding barriers to mental health services. Black individuals in SF are hospitalized at higher rates than other racial groups,^10^ potentially reflecting inadequate outpatient treatment and a greater need for acute psychiatric care. However, no studies to date have examined how hospital characteristics influence the use of physical restraints in EDs across SF.

In this exploratory study, we evaluate differences in physical restraint application on ED mental health patients at two hospitals in SF—a publicly funded trauma center and an academic tertiary care center. While our data focus on SF, the structural issues driving these disparities are not unique to the city. Given the inclusion of both an academic and a public hospital, our findings may offer insights generalizable to the health systems both locally and nationally.^11^

## Methods

### Study design and setting

This retrospective cross-sectional study examined adult patients who sought emergency mental health care at two EDs. For the University of California, San Francisco Parnassus Hospital (UCSF), patient encounters between January 1, 2019, and December 31, 2020, were included, while for the public hospital, Zuckerberg San Francisco General Hospital (ZSFG), data were collected from August 3, 2019, to December 31, 2020, following the transition to an electronic medical record (EMR) system. The UCSF Institutional Review Board approved this study, and reporting adheres to STROBE guidelines.^12^

UCSF is an urban, academic center located in central SF, with an annual ED census of over 40,000 visits. All patients presenting with mental health-related concerns are evaluated by an emergency medicine provider, and, if needed, a psychiatry resident is available 24/7 for consultation under attending psychiatrist oversight.

ZSFG is the only level one trauma center in SF, serving a predominantly underserved, urban population. The ED sees over 60,000 annual visits.^13^ At ZSFG, medically stable patients presenting solely for mental health concerns may be directly transported to the Psychiatric Emergency Services unit by Emergency Medical Services (EMS) and bypass the main ED. This practice was suspended during the COVID-19 pandemic to ensure all patients were screened for COVID-19 before receiving care.

### Community-based participatory research

We partnered with the Rafiki Coalition for Health and Wellness, a community organization with strong roots in SF’s Black community. In partnership with their executive director, we identified three additional individuals to serve on our Community Advisory Board (CAB). CAB members were selected based on their lived experience, interest in improving mental health care in the community, and availability to participate. CAB meetings were conducted throughout the study, with members shaping the conceptual framework, variable selection, and result interpretation.

### Selection of participants

This study included all adults who presented to the ED who met one of the following criteria: (1) receipt of a psychiatric consultation in the ED, (2) medical admission following a suicide attempt, or (3) placement on an ED hold. An ED hold is a temporary measure placed by ED staff on patients deemed a potential risk to themselves or others, or to be unable to care for themselves due to severe mental illness. This designation remains in place while patients await assessment to determine the need for involuntary psychiatric detention.

### Measures

All measures were extracted from the EMR. The primary outcome was a binary designation of physical restraint application, determined by the presence of an EMR order for physical restraints. All patients placed in restraints must have a corresponding EMR order placed at the time of restraint or immediately after.

The primary exposure variable was patient race, as documented in the EMR. At both hospitals, it is standard for patients to self-select race, ethnicity, and other demographic descriptors for inclusion in the EMR when possible. If a patient has been seen previously, these descriptors are carried forward to subsequent encounters. EMR categories of race included American Indian or Alaskan Native, Asian, Black, declined to answer, Native Hawaiian or Pacific Islander, Other, Other Pacific Islander, Unknown, and White. For analysis, racial groups were consolidated into four categories: Asian, Black, White, and Other, due to limited sample sizes in some groups. Ethnicity was documented as Hispanic or non-Hispanic. Alternative methods of reporting race and ethnicity were explored, including not combining groups and incorporating race and ethnicity together (e.g., non-Hispanic Black, Hispanic White).

Models were adjusted for demographic, socioeconomic, and health risk factors likely to influence risk of restraint: age, sex, insurance type, housing status, time of ED arrival, method of ED arrival, and documented history of mental health or substance use-related diagnoses in the EMR. Hospital type accounted for system-level factors.

### Data analysis

Descriptive analyses consisted of frequency distributions of study sample characteristics using chi-square test of homogeneity to assess differences between patients who were and were not restrained. Primary analyses used multivariable logistic regression to estimate the association between patient race and restraint, adjusted for covariates using clustered standard errors to account for repeated observations of patients with multiple encounters. Stepwise and nested regressions assessed if covariates were independently associated with the outcome (*p* < 0.05). Because patient sex and site location may modify the primary association between race and risk of restraint, we conducted two secondary analyses: (1) stratified models by sex and (2) stratified models by hospital. All data analysis was completed using Stata 17.0 (StataCorp, version 17, 2021. College Station, TX).

## Results

### Patient demographics

We identified 6,631 encounters across both sites during the study period: 3,244 (48.9%) at ZSFG and 3,387 (51.1%) at UCSF, of which 1,051 (15.8%) had an order for physical restraints. Given no significant difference in results combining race and ethnicity (Supplementary Table S1), we report estimates separately. White race was most common (*n* = 2,864, 43.2%), followed by Other (*n* = 1,609, 24.3%), Black (*n* = 1,394, 21.0%), and Asian (*n* = 764, 11.5%). Within the ‘Other’ category less than 1% identified as American Indian or Alaskan Native, Native Hawaiian or Pacific Islander, or Other Pacific Islander, and the remainder selected ‘Other’. Most individuals identified as not Hispanic/Latino/a (*n* = 5,439, 81.9%) (Table 1). Differences in patient characteristics by hospital can be reviewed in Supplementary Table S2.

**Table 1.** Descriptive Characteristics of Cohort by Restraint Order

	Not restrained (*n* = 5,580)	Restrained (*n* = 1,051)	Total (*n* = 6,631)
Race			
Asian	673 (12.1)	91 (8.7)	764 (11.5)
Black	1,125 (20.2)	269 (25.6)	1,394 (21.0)
White	2,455 (44.0)	409 (38.9)	2,864 (43.2)
Other	1,327 (23.8)	282 (26.8)	1,609 (24.3)
Ethnicity			
Hispanic/Latino/a	834 (14.9)	148 (14.1)	982 (14.8)
Not Hispanic/Latino/a	4,581 (82.1)	851 (81.0)	5,432 (81.9)
Unknown	165 (3.0)	52 (4.9)	217 (3.3)
Sex			
Female	2,124 (38.1)	353 (33.6)	2,477 (37.4)
Male	3,444 (61.7)	692 (65.8)	4,136 (62.4)
Other/Unknown	12 (0.2)	6 (0.6)	18 (0.3)
Age (years)			
<29	995 (17.8)	133 (12.7)	1,128 (17.0)
30–39	1,180 (21.1)	185 (17.6)	1,365 (20.6)
40–49	1,412 (25.3)	352 (33.5)	1,764 (26.6)
50–59	1,113 (19.9)	210 (20.0)	1,323 (20.0)
60+	880 (15.8)	171 (16.3)	1,051 (15.8)
Time of arrival			
7A–3P	2,078 (37.2)	413 (39.3)	2,491 (37.6)
3P–11P	2,414 (43.3)	438 (41.7)	2,852 (43.0)
11P–7A	1,088 (19.5)	200 (19.0)	1,288 (19.4)
Insurance type			
Commercial	1,017 (18.2)	101 (9.6)	1,118 (16.9)
Medicaid	2,505 (44.9)	497 (47.3)	3,002 (45.3)
Medicare	971 (17.4)	176 (16.7)	1,147 (17.3)
Self-Pay	416 (7.5)	119 (11.3)	535 (8.1)
Unknown	671 (12.0)	158 (15.0)	829 (12.5)
Housing status			
Housed	3,821 (68.5)	626 (59.6)	4,447 (67.1)
Unhoused	1,597 (28.6)	387 (36.8)	1,984 (29.9)
Other	162 (2.9)	38 (3.6)	200 (3.0)
Mental health disorder^a^			
Absent	4,002 (71.7)	858 (81.6)	4,860 (73.3)
Present	1,578 (28.3)	193 (18.4)	1,771 (26.7)
Substance use disorder^a^			
Absent	5,114 (91.6)	976 (92.9)	6,090 (91.8)
Present	466 (8.4)	75 (7.1)	541 (8.2)
Arrival method			
Ambulance	2,585 (46.3)	695 (66.1)	3,280 (49.5)
Self	2,292 (41.1)	242 (23.0)	2,534 (38.2)
Police	390 (7.0)	67 (6.4)	457 (6.9)
Other	226 (4.1)	29 (2.8)	255 (3.8)
Unknown	87 (1.6)	18 (1.7)	105 (1.6)
Hospital			
ZSFG	2,621 (47.0)	623 (59.3)	3,244 (48.9)
UCSF	2,959 (53.0)	428 (40.7)	3,387 (51.1)

a One or more prior International Classification of Diseases (ICD) codes present in chart prior to presentation.

UCSF, University of California, San Francisco Parnassus Hospital; ZSFG, Zuckerberg San Francisco General Hospital.

### Physical restraint use

At the ZSFG ED, 19.2% (*n* = 623) of patients were restrained, whereas 12.6% (*n* = 428) were restrained at the UCSF ED. Among the full study cohort, 19.3% (*n* = 269) of Black patients, 19.9% (*n* = 352) of patients aged 40–49, 22.2% (*n* = 119) of patients who were Self-Pay, 19.5% (*n* = 387) of people experiencing homelessness, and 21.2% (*n* = 695) of those arriving by ambulance were restrained.

### Regression analyses

Asian patients had the lowest prevalence of restraints and served as the reference group in multivariable regression analyses. Black patients had the highest odds of being restrained (OR 1.77, 95% confidence interval [CI] 1.37–2.28) (Table 2), an association that attenuated but persisted after controlling for ethnicity, sex, age, time of arrival, insurance type, housing status, preexisting mental health or substance use disorder, arrival method, and hospital (adjusted odds ratio [aOR] 1.54, 95% CI 1.16–2.05) (Table 2). When stratified by hospital, Black patients had significantly higher odds of restraint at the UCSF ED (aOR 1.77, 95% CI 1.12–2.79) (Table 3). At the ZSFG ED, Black patients had higher odds of restraint (aOR 1.44, 95% CI 0.99–2.09), though not statistically significant, while Other Race had higher odds (aOR 1.53, 95% CI 1.02–2.30) of restraint (Table 3).

**Table 2.** Univariate and Multivariable Logistic Regression Model Predicting Restraint Use, Combined Hospital Analysis^a^

	Univariate odds ratio	95% CI	*p* value	Adjusted odds ratio	95% CI	*p* value
Race						
Asian	Reference			Reference		
Black	1.77	[1.37, 2.28]	**<0.001**	1.54	[1.16, 2.05]	**0.003**
White	1.23	[0.97, 1.57]	0.092	1.20	[0.92, 1.56]	0.186
Other	1.57	[1.22, 2.03]	**<0.001**	1.42	[1.04, 1.94]	**0.026**
Ethnicity						
Hispanic/Latino/a	0.96	[0.79, 1.15]	0.636	0.87	[0.68, 1.11]	0.270
Non-Hispanic/Latino/a	Reference			Reference		
Sex						
Female	Reference			Reference		
Male	1.21	[1.05,1.39]	**0.008**	1.05	[0.89, 1.22]	0.578
Age						
<29	0.69	[.54, 0.88]	**0.003**	0.68	[0.52, 0.90]	**0.008**
30–39	0.81	[0.64, 1.01]	0.062	0.99	[0.77, 1.28]	0.964
40–49	1.28	[1.05, 1.57]	**0.015**	1.33	[1.06, 1.66]	**0.012**
50–59	0.97	[0.78, 1.21]	0.793	0.91	[0.72, 1.16]	0.462
60+	Reference			Reference		
Time of arrival						
7A–3P	Reference			Reference		
3P–11P	0.91	[0.79, 1.06]	0.233	0.94	[0.81, 1.11]	0.478
11P–7A	0.92	[0.77, 1.11]	0.406	0.87	[0.71, 1.06]	0.159
Insurance type						
Commercial	Reference			Reference		
Medicaid	2.00	[1.59, 2.50]	**<0.001**	1.24	[0.96, 1.61]	0.103
Medicare	1.83	[1.41, 2.37]	**<0.001**	1.42	[1.04, 1.94]	**0.026**
Self-Pay	2.88	[2.16, 3.84]	**<0.001**	1.49	[1.06, 2.09]	**0.020**
Unknown	2.37	[1.81, 3.10]	**<0.001**	1.35	[0.99, 1.86]	0.060
Housing status						
Housed	Reference			Reference		
Unhoused	1.48	[1.29, 1.70]	**<0.001**	1.12	[0.95, 1.31]	0.184
Mental health disorder						
Absent	Reference			Reference		
Present	0.57	[0.48, 0.67]	**<0.001**	0.69	[0.57, 0.83]	**<0.001**
Substance use disorder						
Absent	Reference			Reference		
Present	0.84	[0.65, 1.09]	0.187	1.09	[0.82, 1.44]	0.574
Arrival method						
Self	Reference			Reference		
Ambulance	2.55	[2.18, 2.98]	**<0.001**	2.35	[1.99, 2.78]	**<0.001**
Other	1.54	[1.21, 1.95]	**<0.001**	1.3	[1.01, 1.68]	**0.046**
Hospital						
ZSFG	Reference			Reference		
UCSF	0.61	[0.53, 0.70]	**<0.001**	0.63	[0.54, 0.74]	**<0.001**
Intercept				0.13	[0.09, 0.19]	**<0.001**

a Bolded *p* values significant at 95% confidence level.

The adjusted multivariate model included race, ethnicity, sex, age, time of arrival, insurance type, housing status, mental health disorder, substance use disorder, arrival method and hospital.

CI, confidence interval; UCSF, University of California, San Francisco Parnassus Hospital; ZSFG, Zuckerberg San Francisco General Hospital.

**Table 3.** Multivariable Logistic Regression Model of Restraint Use by Hospital^a^

	ZSFG			UCSF		
	Adjusted odds ratio	95% CI	*p* value	Adjusted odds ratio	95% CI	*p* value
Race						
Asian	Reference			Reference		
Black	1.44	[0.99, 2.09]	0.055	1.77	[1.12, 2.79]	**0.015**
White	1.23	[0.86, 1.76]	0.250	1.23	[0.81, 1.85]	0.330
Other	1.53	[1.02, 2.30]	**0.038**	1.27	[0.78, 2.09]	0.340
Ethnicity						
Hispanic/Latino/a	0.76	[0.55, 1.04]	0.084	1.07	[0.73, 1.59]	0.723
Non-Hispanic/Latino/a	Reference			Reference		
Sex						
Female	Reference			Reference		
Male	0.91	[0.75, 1.12]	0.395	1.20	[0.94, 1.52]	0.140
Age						
<29	0.81	[0.57, 1.16]	0.256	0.54	[0.35, 0.84]	0.006
30–39	0.98	[0.70, 1.37]	0.897	1.02	[0.68, 1.53]	0.921
40–49	1.33	[1.00, 1.76]	**0.050**	1.34	[0.93, 1.92]	0.112
50–59	0.93	[0.69, 1.26]	0.654	0.84	[0.57, 1.24]	0.383
60+	Reference			Reference		
Time of arrival						
7A–3P	Reference			Reference		
3P–11P	0.91	[0.74, 1.12]	0.385	0.92	[0.72, 1.17]	0.490
11P–7A	0.95	[0.74, 1.22]	0.684	0.71	[0.52, 0.97]	0.033
Insurance						
Commercial	Reference			Reference		
Medicaid	1.07	[0.66, 1.75]	0.772	1.15	[0.83, 1.59]	0.411
Medicare	1.55	[0.91, 2.65]	0.109	0.92	[0.59, 1.44]	0.725
Self-pay	1.25	[0.70, 2.24]	0.456	1.34	[0.86, 2.07]	0.196
Unknown	1.19	[0.71, 1.98]	0.504	1.35	[0.77, 2.35]	0.291
Housing status						
Housed	Reference			Reference		
Unhoused	1.20	[0.99, 1.47]	0.068	0.96	[0.72, 1.26]	0.749
Mental health disorder						
Absent	Reference			Reference		
Present	0.70	[0.54, 0.92]	**0.011**	0.74	[0.55, 0.99]	0.040
Substance use disorder						
Absent	Reference			Reference		
Present	0.91	[0.59, 1.40]	0.663	1.26	[0.86, 1.84]	0.243
Arrival method						
Self	Reference			Reference		
Ambulance	1.40	[1.14, 1.73]	**0.001**	5.83	[4.25, 8.00]	**<0.001**
Other	0.92	[0.69, 1.24]	0.590	2.73	[1.64, 4.54]	**<0.001**
Intercept	0.15	[0.08, 0.27]	**<0.001**	0.05	[0.03, 0.09]	**<0.001**

a Bolded *p* values significant at 95% confidence level.

The adjusted multivariate model included race, ethnicity, sex, age, time of arrival, insurance type, housing status, mental health disorder, substance use disorder, arrival method and hospital.

CI, confidence interval; UCSF, University of California, San Francisco Parnassus Hospital; ZSFG, Zuckerberg San Francisco General Hospital.

### Other covariates of interest

#### Arrival method

Many patients arrived by EMS (*n* = 3,280, 49.5%) (Table 1). In univariable analyses, those arriving by EMS had 2.55 (95% CI 2.18–2.98) the odds of restraint compared with those who self-presented (Supplementary Table S3). Arriving by ambulance was the single strongest predictor of restraint use in combined hospital multivariable analysis (aOR 2.35, 95% CI 1.99–2.78) (Table 2) and stratified analyses (UCSF aOR 5.83, 95% CI 4.25–8.00 and ZSFG aOR 1.40, 95% CI 1.14–1.73) (Table 3).

#### Comorbidity

Overall, 26.7% (*n* = 1,772) of individuals had a diagnosed comorbid mental health disorder, and 8.2% (*n* = 541) overall had a diagnosed substance use disorder. In multivariable combined-hospital analyses, presence of a mental health disorder was associated with lower odds of restraint (aOR 0.69, 95% CI 0.57–0.83, Table 2). Presence of a substance use disorder diagnosis was not associated with restraint in either univariable or multivariable analyses.

#### Sex

In combined hospital multivariable regression analysis stratified by sex, we found that the association between Black race and restraint use held for both male (aOR 1.46, 95% CI 1.02–2.11) and female (aOR 1.74, 95% CI 1.10–2.75) patients. Males less than 29 years of age had lower odds of restraint compared with those 60 or older (aOR 0.67, 95% CI 0.48–0.95), and those 40–49 had higher odds of restraint (aOR 1.37, 95% CI 1.05–1.79), but these associations were not significant among female patients (Supplementary Table S3).

## Discussion

To our knowledge this is the first study to compare variations in the use of physical restraints among adults specifically with mental health-related ED visits in a public vs. private medical institution within the same city. Our findings indicate a disproportionate burden of restraint use among Black-identifying patients presenting to the ED with mental health concerns. This effect remained significant at the private, academic hospital and was just slightly below the significance threshold at the public hospital. We acknowledge the findings for Black-identifying patients were not significant at the public hospital; however, given that the pooled estimate as well as the estimate in the private hospital were significant, and the lower bound of the CI was 0.99 with an upper greater than 2, it is likely the nonsignificant finding in the public hospital was due to chance. Further work to explore this relationship with a larger sample size at the public hospital may be needed to clarify the association.

In the combined hospital analysis, patients aged 40–49, insured by Medicare, or transported to the hospital via ambulance had increased odds of being restrained. Notably, ethnicity was not a significant factor in restraint use across any analysis. The overall similarity in findings between the two hospital settings suggests that hospital type is not a primary driver of racial disparities in restraint use.

Our results align with a recent systematic review of restraint use among all comers in EDs, which found higher odds of restraint among Black individuals, whereas Hispanic patients had a lower prevalence.^6^ However, a recent study specifically examining ED restraints use reported significantly higher odds of restraint use among non-Hispanic Black and Hispanic-identifying patients than non-Hispanic White individuals.^14^ Consistent with prior research, we found that patients with non-private insurance—including Medicaid, Self-Pay, public insurance, or “lack of private insurance”—had higher odds of restraint use.^15^

Although numerous studies have demonstrated a consistent association between mental health diagnoses and increased restraint use,^14–16^ we observed a decrease in the odds of restraint use among patients with a documented mental health diagnosis. One possible explanation is that ED providers, when treating patients with known mental illness and access to their EMR history, may be better able to anticipate patient behavior and thus less inclined to use restraints during crises. Conversely, patients with undiagnosed mental illness may be perceived as more unpredictable or threatening, increasing the likelihood of restraint use. In addition, patients in our cohort who have previously accessed mental health care services within our health system may be less likely to require ED ‘crisis care’ than someone without regular mental health care. Future research should explore differences in restraint use based on whether a patient has a known versus unknown mental health diagnosis at the time of ED presentation.

The mechanisms leading to the differential application of restraints on Black patients in EDs have yet to be definitively described, though the associations described above are hypothesis generating. Patients with mental health crises may present with acute agitation, yet standardized protocols for management are inconsistently applied.^17^ Prior studies indicate that ED staff harbor implicit racial biases,^18–21^ which can influence critical decision-making, particularly in high-risk situations. When managing agitated patients, these biases may contribute to differential treatment.^22^

Institutional policies related to staffing may also play a role. Critical care nurses have described how staffing ratios and physical and psychological staff exhaustion may contribute to restraint use,^23^ and EDs face similar staffing shortages.^24^ Future studies by this group include qualitative work with community members and ED staff to better elucidate these mechanisms.

Patients arriving by EMS had higher odds of being restrained compared with those who self-presented to the ED. This may reflect a greater severity of illness among patients arriving via EMS. Alternatively, prehospital interactions with bystanders, police, EMS, and other first responders may exacerbate agitation, leading to increased restraint use upon ED arrival. Other research has shown that police transport to the ED mediates the relationship between Black race and physical restraint use.^25^ Future research should investigate whether prehospital restraint placement influences subsequent ED restraint use. Additional research is needed to understand demographic patterns in EMS utilization for mental health emergencies and to identify potential disparities in prehospital care that may contribute to differential restraint use in the ED.

### Limitations

Our primary exposure variable was race, not experiences of racism, and these two constructs are not interchangeable.^26,27^ Although racism is a fundamental driver of racial disparities in ED restraint use, its measurement within health systems remains challenging, limiting our ability to directly quantify its impact. As a result, our findings are based on available data and require inferential interpretation. Because health-promoting opportunities are influenced by both race and socioeconomic status (SES),^28^ we examined the independent role of race while controlling for SES by incorporating variables like insurance and housing status into our models. However, residual confounding likely remains. In forthcoming work, we aim to address this by incorporating qualitative data to better understand the underlying mechanisms behind the identified differences. We hypothesize that interpersonal, institutional, and structural racism may play a significant role.

Our study was limited by a shorter data collection period at the public hospital compared with the academic hospital, and primarily during the fall and winter months. Seasonal variations in mental health symptoms^29^ and in the use of restraint/seclusion^30^ have been documented, and these temporal factors may have influenced our results. Furthermore, data were included during the time of the COVID-19 emergency stay-at-home measures, a period marked by significant increase in negative mental health symptoms. The potential impact of this rise in societal mental unwellness on our findings is unclear and may limit the generalizability of our conclusions.

We did not assess the appropriateness of restraint use in this study because the American Association of Emergency Psychiatry published Project Best Practices in the Evaluation and Treatment of Agitation (BETA),^31^ which strongly recommends against restraint use whenever possible. According to these guidelines, any deviation (i.e., restraint use) should result in an equally distributed consequence or differences explained due to chance, which was not the case for Black patients in our study.

### Health equity implications

A lack of evidence illustrating a broader impact on other patients of color suggests that a general racial equity framework may be insufficient to explain our findings. Instead, we propose that the racially differentiated use of physical restraints is a direct consequence of anti-Black racism. Anti-Black racism refers to dehumanization of Black individuals grounded in colonialism, enslavement, and ongoing subjugation of Black communities. Previous literature underscores the importance of explicitly naming this type of racism.^32^ Engaging in discussions about anti-Black racism is essential for advancing health equity. Disparities in psychiatric care and other fields of medicine disproportionately affect Black individuals due to the historical and enduring impacts of anti-Black racism. For example, Black individuals are more likely to be placed on involuntary legal holds^33^ and to receive inpatient psychiatric consultations,^7^ but are less likely to receive outpatient psychiatric care,^34^ even following acute psychiatric hospitalization.^35,36^ Restraint use is linked to a reduced likelihood of successful mental health follow-up.^37^ Insufficient engagement in timely preventative and therapeutic care may increase the likelihood of acute presentations due to the deterioration of underlying mental and physical health conditions.^7^ In this context, disproportionate use of physical restraints exacerbates the mental health equity crisis for Black patients.

Health systems magnify broader societal dynamics, and a greater focus on both the mechanisms that perpetuate bias and those that foster equity can provide valuable insights into how to reduce disparities beyond the medical context. Our study highlights the critical need for targeted mental health needs assessments, paired with interventions that are informed by an understanding of the broader impact of structural racism. There is a critical need in health to account for the historical trauma experienced by Black communities, both within and beyond medical care, and to avoid causing new trauma through coercive action. Through community partnerships, health systems must improve care delivery so that Black patients feel safe, welcome, and empowered to seek mental health services. These goals for health systems align with medicine’s objectives to alleviate suffering and guide healing and will require a commitment from health care leaders to fight anti-Black racism. This study underscores the need to implement reforms in the emergency mental health care system for more racially equitable and patient-centered care.

## Authors’ Contributions

D.J.: Data curation, formal analysis, software, writing original draft, and writing—review and editing. L.A.: Data curation, formal analysis, and writing original draft. A.H.: Formal analysis, supervision, writing original draft, and writing-review and editing. K.W.: Formal analysis, supervision, writing original draft, and writing-review and editing. M.L.: Conceptualization, funding acquisition, methodology, project administration, resources, writing—review and editing, and supervision. A.B.: Conceptualization, project administration, and resources. V.J.: Conceptualization, methodology, and writing—review and editing. T.H.: Conceptualization, methodology, and writing—review and editing. L.A.: Project administration, and writing—review and editing. S.W.: Project administration, and writing—review and editing. R.D.: Conceptualization, funding acquisition, methodology, and writing—review and editing. A.R.H.: Conceptualization, funding acquisition, methodology, writing original draft, writing—review and editing, and supervision. W.M.: Conceptualization, funding acquisition, methodology, writing—review and editing, and supervision. M.D.T.: Methodology, data curation, formal analysis, and software. V.E.: Conceptualization, funding acquisition, methodology, project administration, writing original draft, writing—review and editing, and supervision.

## Supplemental Material

sj-docx-1-heq-10.1177_24731242251394114 — Supplemental material for Comparing Disparate Application of Physical Restraints on Patients Seeking Emergency Mental Health Care at Two San Francisco Emergency Departments Supplemental material, sj-docx-1-heq-10.1177_24731242251394114 for Comparing Disparate Application of Physical Restraints on Patients Seeking Emergency Mental Health Care at Two San Francisco Emergency Departments by

## Abbreviations

aOR: Adjusted odds ratio
BETA: Best practices in the evaluation and treatment of agitation
CAB: Community Advisory Board
ED: Emergency department
EMR: Electronic medical record
EMS: Emergency medical services
SF: San Francisco
USA: United States of America
UCSF: University of California San Francisco
ZSFG: Zuckerberg San Francisco General

## References

[1] Theriault KM, Rosenheck RA, Rhee TG. Increasing emergency department visits for mental health conditions in the United States. J Clin Psychiatry, 2020; 81(5):20m13241; doi: 10.4088/JCP.20m13241 32726001

[2] Schwartz RC, Blankenship DM. Racial disparities in psychotic disorder diagnosis: A review of empirical literature. World J Psychiatry, 2014; 4(4):133–140; doi: 10.5498/wjp.v4.i4.133 PMC4274585 25540728

[3] Panchal N, Hill L, Artiga S, et al. Racial and Ethnic Disparities in Mental Health Care: Findings from the KFF Survey of Racism, Discrimination and Health. 2024.

[4] Hartz SM, Pato CN, Medeiros H, et al.; Genomic Psychiatry Cohort Consortium. Comorbidity of severe psychotic disorders with measures of substance use. JAMA Psychiatry, 2014; 71(3):248–254; doi: 10.1001/jamapsychiatry.2013.3726 PMC4060740 24382686

[5] Substance Abuse and Mental Health Services Administration. Key Substance Use and Mental Health Indicators in the United States: Results from the 2021 National Survey on Drug Use and Health. In: HHS Publication No PEP22-07-01-005, NSDUH Series H-57. Substance Abuse and Mental Health Services Administration; 2022.

[6] Eswaran V, Molina MF, Hwong AR, et al. Racial disparities in emergency department physical restraint use: A systematic review and meta-analysis. JAMA Intern Med, 2023; 183(11):1229–1237; doi: 10.1001/jamainternmed.2023.4832 PMC10520842 37747721

[7] Zambrano J, Celano CM, Onyeaka H, et al. Ethnoracial disparities in care on a consultation-liaison service at an academic hospital. Gen Hosp Psychiatry, 2024; 86:50–55; doi: 10.1016/j.genhosppsych.2023.11.010 PMC10843593 38070241

[8] Moore ELI, Montojo N, Mauri N. Roots, Race, and Place: A History of Racially Exclusionary Housing in the San Francisco Bay Area. Haas Institute for a Fair and Inclusive Society, University of California, Berkeley. 2019. Available from: https://belonging.berkeley.edu/rootsraceplace

[9] De Sousa T, Henry M. The 2024 Annual Homelessness Assessment Report (AHAR to Congress) Part 1: Point-In-Time Estimates of Homelessness, The US Department of Housing and Urban Development Office of Community Planning and Development, December 2024.

[10] Stookey J, Ohtake R, Hecht H.Mental Health of MCAH Populations in San Francisco, San Francisco Department of Public Health. 2021. Available from: https://www.sf.gov/sites/default/files/2024-11/8-MCAH_mental_health_2021.pdf [Accessed November 3, 2025].

[11] Yearby R, Clark B, Figueroa JF. Structural racism in historical and modern US health care policy. Health Aff (Millwood), 2022; 41(2):187–194; doi: 10.1377/hlthaff.2021.01466 35130059

[12] von Elm E, Altman DG, Egger M, et al.; STROBE Initiative. The Strengthening the Reporting of Observational Studies in Epidemiology (STROBE) statement: Guidelines for reporting observational studies. J Clin Epidemiol, 2008; 61(4):344–349; doi: 10.1016/J.JCLINEPI.2007.11.008 18313558

[13] Zuckerberg San Francisco General Hospital and Trauma Center, San Francisco Department of Public Health, San Francisco Health Network. Zuckerberg San Francisco General Hospital and Trauma Center GY 2022–2023 Annual Report: San Francisco, CA; n.d.

[14] Tartak JAC, Brisbon N, Wilkie S, et al. Racial and ethnic disparities in emergency department restraint use: A multicenter retrospective analysis. Academic Emergency Medicine, 2021; 28:957–965; doi: 10.1111/ACEM.14327 34533261

[15] Wong AH, Whitfill T, Ohuabunwa EC, et al. Association of race/ethnicity and other demographic characteristics with use of physical restraints in the emergency department. JAMA Netw Open, 2021; 4(1):e2035241. 10.1001/jamanetworkopen.2020.35241 PMC7835716 33492372

[16] Tolliver DG, Markowitz MA, Obiakor KE, et al. Characterizing racial disparities in emergency department pediatric physical restraint by sex and age. JAMA Pediatr, 2023; 177(9):972–975; doi: 10.1001/jamapediatrics.2023.2300 PMC10352924 37459087

[17] Downey LVA, Zun LS, Gonzales SJ. Frequency of alternative to restraints and seclusion and uses of agitation reduction techniques in the emergency department. Gen Hosp Psychiatry, 2007; 29(6):470–474; doi: 10.1016/J.GENHOSPPSYCH.2007.07.006 18022038

[18] Zeidan AJ, Khatri UG, Aysola J, et al. Implicit bias education and emergency medicine training: Step one? Awareness. AEM Educ Train, 2019; 3(1):81–85; doi: 10.1002/aet2.10124 PMC6339553 30680351

[19] Dehon E, Weiss N, Jones J, et al. A systematic review of the impact of physician implicit racial bias on clinical decision making. Acad Emerg Med, 2017; 24(8):895–904; doi: 10.1111/acem.13214 28472533

[20] Green AR, Carney DR, Pallin DJ, et al. Implicit bias among physicians and its prediction of thrombolysis decisions for black and white patients. J Gen Intern Med, 2007; 22(9):1231–1238; doi: 10.1007/s11606-007-0258-5 PMC2219763 17594129

[21] Boley S, Sidebottom A, Vacquier M, et al. Racial differences in stigmatizing and positive language in emergency medicine notes. J Racial Ethn Health Disparities, 2024; doi: 10.1007/s40615-024-02080-3 38980524

[22] Jin RO, Anaebere TC, Haar RJ. Exploring bias in restraint use: Four strategies to mitigate bias in care of the agitated patient in the emergency department. Acad Emerg Med, 2021; 28(9):1061–1066; doi: 10.1111/acem.14277 33977591

[23] Teece A, Baker J, Smith H. Understanding the decision-making of critical care nurses when restraining a patient with psychomotor agitation secondary to hyperactive delirium: A ‘Think Aloud’ study. J Clin Nurs, 2022; 31(1–2):121–133; doi: 10.1111/jocn.15889 34056784

[24] Hodgson NR, Kwun R, Gorbatkin C, et al.; ACEP Emergency Medicine Practice Committee. Emergency department responses to nursing shortages. Int J Emerg Med, 2024; 17(1):51; doi: 10.1186/s12245-024-00628-y PMC10996074 38580916

[25] Chang-Sing E, Smith CM, Gagliardi JP, et al. Racial and ethnic disparities in patient restraint in emergency departments by police transport status. JAMA Netw Open, 2024; 7(2):e240098; doi: 10.1001/jamanetworkopen.2024.0098 PMC10882414 38381433

[26] Dean LT, Thorpe RJ. What structural racism is (or Is Not) and how to measure it: Clarity for public health and medical researchers. Am J Epidemiol, 2022; 191(9):1521–1526; doi: 10.1093/aje/kwac112 PMC9437815 35792088

[27] Adkins-Jackson PB, Chantarat T, Bailey ZD, et al. Measuring structural racism: A guide for epidemiologists and other health researchers. Am J Epidemiol, 2022; 191(4):539–547; doi: 10.1093/aje/kwab239 PMC9077112 34564723

[28] Kawachi I, Daniels N, Robinson DE. Health disparities by race and class: Why both matter. Health Aff (Millwood), 2005; 24(2):343–352; doi: 10.1377/hlthaff.24.2.343 15757918

[29] Geoffroy PA, Bellivier F, Scott J, et al. Seasonality and bipolar disorder: A systematic review, from admission rates to seasonality of symptoms. J Affect Disord, 2014; 168:210–223; doi: 10.1016/j.jad.2014.07.002 25063960

[30] Kuivalainen S, Vehviläinen-Julkunen K, Louheranta O, et al. Seasonal variation of hospital violence, seclusion and restraint in a forensic psychiatric hospital. Int J Law Psychiatry, 2017; 52:1–6; doi: 10.1016/j.ijlp.2017.05.004 28506820

[31] Holloman GH, Zeller SL. Overview of project BETA: Best practices in evaluation and treatment of agitation. West J Emerg Med, 2012; 13(1):1–2; doi: 10.5811/WESTJEM.2011.9.6865 PMC3298232 22461914

[32] Eisape A, Nogueira A. See change: Overcoming anti-black racism in health systems. Front Public Health, 2022; 10:895684; doi: 10.3389/fpubh.2022.895684 PMC9245034 35784218

[33] Shea T, Dotson S, Tyree G, et al. Racial and ethnic inequities in inpatient psychiatric civil commitment. Psychiatr Serv, 2022; 73(12):1322–1329; doi: 10.1176/appi.ps.202100342 35959533

[34] Racial-Ethnic Disparities in Outpatient Mental Health Care in the United States. n.d; doi: 10.1176/appi.ps.20220365 36597696

[35] Fontanella CA, Warner LA, Steelesmith DL, et al. Association of timely outpatient mental health services for youths after psychiatric hospitalization with risk of death by suicide. JAMA Netw Open, 2020; 3(8):e2012887; doi: 10.1001/jamanetworkopen.2020.12887 PMC7420244 32780122

[36] Smith TE, Corbeil T, Wall MM, et al. Community, hospital, and patient factors contributing to ethnoracial disparities in follow-up after psychiatric hospitalization. Psychiatr Serv, 2023; 74(7):684–694; doi: 10.1176/appi.ps.20220110 PMC10329986 36651116

[37] Currier GW, Walsh P, Lawrence D. Physical restraints in the emergency department and attendance at subsequent outpatient psychiatric treatment. J Psychiatr Pract, 2011; 17(6):387–393; doi: 10.1097/01.PRA.0000407961.42228.75 22108395

