# Supplementary material for: Comparing Disparate Application of Physical Restraints on Patients Seeking Emergency Mental Health Care at Two San Francisco Emergency Departments
Source: Health Equity. 2026 Mar 4;10:24731242251394114. doi: 10.1177/24731242251394114 (PMC13724980; doi:10.1177/24731242251394114)
Supplement: Supplementary file 1 [file sj-docx-1-heq-10.1177_24731242251394114.docx]

Supplemental Table 1

Supplemental Table 1: Logistic regression model predicting restraint use at Parnassus and ZSFG*

|  | Total | Odds Ratio | 95% CI | p-value |
| --- | --- | --- | --- | --- |
| **Race** |  |  |  |  |
| Asian, Hispanic/Latino/a | 17 (0.3%) | 1.57 | [0.43, 5.74] | 0.499 |
| Asian, Not Hispanic/Latino/a | 739 (11.5%) | Reference |  |  |
| Black, Hispanic/Latino/a | 27 (0.4%) | 1.89 | [0.67, 5.30] | 0.229 |
| Black Not Hispanic/Latino/a | 1360 (21.2%) | 1.55 | [1.16, 2.08] | **0.003** |
| White Hispanic/Latino/a | 186 (2.9%) | 1.30 | [0.80, 2.12] | 0.290 |
| White Not Hispanic/Latino/a | 2649 (41.3%) | 1.20 | [0.91, 1.57] | 0.191 |
| Other Hispanic/Latino/a | 752 (11.7%) | 1.17 | [0.84, 1.62] | 0.348 |
| Other Not Hispanic/Latino/a | 684 (10.7%) | 1.55 | [1.13, 2.14] | **0.007** |
| **Sex** |  |  |  |  |
| Female | 2477 (37.5%) | Reference |  |  |
| Male | 4136 (62.5%) | 1.05 | [0.89, 1.22] | 0.580 |
| **Age** |  |  |  |  |
| <29 | 1128 (17.0%) | 0.69 | [0.52, 0.90] | **0.008** |
| 30-39 | 1365 (20.6%) | 0.99 | [0.77, 1.27] | 0.927 |
| 40-49 | 1764 (26.6%) | 1.32 | [1.06, 1.65] | **0.014** |
| 50-59 | 1323 (20.0%) | 0.91 | [0.72, 1.16] | 0.451 |
| 60+ | 1051 (15.8%) | Reference |  |  |
| **Time of Arrival** |  |  |  |  |
| 7A-3P | 2491 (37.6%) | Reference |  |  |
| 3P-11P | 2852 (43.0%) | 0.94 | [0.80, 1.10] | 0.457 |
| 11P-7A | 1288 (19.4%) | 0.87 | [0.71, 1.05] | 0.151 |
| **Insurance Type** |  |  |  |  |
| Commercial | 1118 (16.9%) | Reference |  |  |
| Medicaid | 3002 (45.3%) | 1.25 | [0.96, 1.62] | 0.099 |
| Medicare | 1147 (17.3%) | 1.42 | [1.04, 1.94] | **0.026** |
| Self-pay | 535 (8.1%) | 1.49 | [1.06, 2.09] | **0.020** |
| Unknown | 829 (12.5%) | 1.36 | [0.99, 1.87] | 0.057 |
| **Housing Status** |  |  |  |  |
| Housed | 4447 (69.1%) | Reference |  |  |
| Unhoused | 1984 (30.9%) | 1.11 | [0.95, 1.31] | 0.199 |
| **Mental Health Disorder** |  |  |  |  |
| Absent | 4860 (73.3%) | Reference |  |  |
| Present | 1771 (26.7%) | 0.68 | [0.56, 0.83] | **<.001** |
| **Substance Use Disorder** |  |  |  |  |
| Absent | 6090 (91.8%) | Reference |  |  |
| Present | 541 (8.2%) | 1.09 | [0.82, 1.45] | 0.548 |
| **Arrival Method** |  |  |  |  |
| Self | 3280 (49.5%) | Reference |  |  |
| Ambulance | 2534 (38.2%) | 2.36 | [1.99, 2.79] | **<.001** |
| Other | 817 (12.3%) | 1.29 | [1.00, 1.67] | 0.053 |
| **Hospital** |  |  |  |  |
| SFGH | 3244 (48.9%) | Reference |  |  |
| Parnassus | 3387 (51.1%) | 0.63 | [0.54, 0.74] | **<.001** |
|  |  |  |  |  |
| **Intercept** |  | 0.09 | [0.06, 0.13] | **<.001** |

*Bolded p values significant at 95% confidence level

| Supplemental Table 2  **Supplemental Table 2**: Descriptive Statistics for each covariate by hospital | | | | | | |
| --- | --- | --- | --- | --- | --- | --- |
|  | Hospital | | | | | |
|  | ZSFG (*n*=3244) | | UCSF (*n*=3387) | | Total (*n*=6,631) | |
| **Race** |  |  |  | n (%) |  |  |
| Asian | 341 | (10.5) | 425 | (12.5) | 766 | (11.5) |
| Black | 808 | (24.8) | 589 | (17.4) | 1397 | (21.0) |
| White | 1229 | (37.8) | 1638 | (48.4) | 2867 | (43.2) |
| Other | 877 | (26.9) | 735 | (21.7) | 1612 | (24.3) |
| **Ethnicity** |  |  |  |  |  |  |
| Hispanic/Latino/a | 579 | (17.8) | 407 | (12.0) | 986 | (14.8) |
| Not Hispanic/Latino/a | 2588 | (79.5) | 2851 | (84.2) | 5439 | (81.9) |
| Unknown | 88 | (2.7) | 129 | (3.8) | 217 | (3.3) |
| **Sex** |  |  |  |  |  |  |
| Female | 1061 | (32.6) | 1418 | (41.9) | 2479 | (37.3) |
| Male | 2186 | (67.2) | 1959 | (57.8) | 4145 | (62.4) |
| Other/Unknown | 8 | (0.2) | 10 | (0.3) | 18 | (0.3) |
| **Age (years)** |  |  |  |  |  |  |
| <29 | 471 | (14.5) | 657 | (19.4) | 1128 | (17.0) |
| 30-39 | 572 | (17.6) | 798 | (23.6) | 1370 | (20.6) |
| 40-49 | 919 | (28.2) | 848 | (25.0) | 1767 | (26.6) |
| 50-59 | 739 | (22.7) | 586 | (17.3) | 1325 | (19.9) |
| 60+ | 554 | (17.0) | 498 | (14.7) | 1052 | (15.8) |
| **Time of Arrival** |  |  |  |  |  |  |
| 7A-3P low risk | 1201 | (36.9) | 1290 | (38.1) | 2491 | (37.5) |
| 3P-11P mid risk | 1400 | (43.0) | 1452 | (42.9) | 2852 | (42.9) |
| 11P-7A high risk | 654 | (20.1) | 645 | (19.0) | 1299 | (19.6) |
| **Insurance Type** |  |  |  |  |  |  |
| Commercial | 139 | (4.3) | 979 | (28.9) | 1118 | (16.8) |
| Medicaid | 1653 | (50.8) | 1349 | (39.8) | 3002 | (45.2) |
| Medicare | 528 | (16.2) | 619 | (18.3) | 1147 | (17.3) |
| Self-Pay | 225 | (6.9) | 310 | (9.2) | 535 | (8.1) |
| Unknown | 710 | (21.8) | 130 | (3.8) | 840 | (12.6) |
| **Housing Status** |  |  |  |  |  |  |
| Housed | 1871 | (57.5) | 2576 | (76.1) | 4447 | (67.0) |
| Unhoused | 1173 | (36.0) | 811 | (23.9) | 1984 | (29.9) |
| Other | 211 | (6.5) | 0 | (0.0) | 211 | (3.2) |
| **Mental Health Disorder*** |  |  |  |  |  |  |
| Absent | 2594 | (79.7) | 2276 | (67.2) | 4870 | (73.3) |
| Present | 661 | (20.3) | 1111 | (32.8) | 1772 | (26.7) |
| **Substance Use Disorder*** |  |  |  |  |  |  |
| Absent | 3059 | (94.0) | 3042 | (89.8) | 6101 | (91.9) |
| Present | 196 | (6.0) | 345 | (10.2) | 541 | (8.1) |
| **Arrival Method**  Ambulance | 1519 | (46.8) | 1761 | (52.0) | 3280 | (49.5) |
| Self | 1157 | (35.7) | 1377 | (40.7) | 2534 | (38.2) |
| Police | 263 | (8.1) | 194 | (5.7) | 457 | (6.9) |
| Other | 204 | (6.3) | 51 | (1.5) | 255 | (3.8) |
| Unknown | 101 | (3.1) | 4 | (0.1) | 105 | (1.6) |
| **Restraint Order Placed** |  |  |  |  |  |  |
| No | 2621 | (80.8) | 2959 | (87.4) | 5580 | (84.2) |
| Yes | 623 | (19.2) | 428 | (12.6) | 1051 | (15.8) |

Supplemental Table 3

Supplemental Table 3: Logistic regression model predicting restraint use at Parnassus and ZSFG stratified by sex

|  | **Male** |  | | |  | **Female** |  |  | |
| --- | --- | --- | --- | --- | --- | --- | --- | --- | --- |
|  | Odds Ratio | 95% CI | | | p-value | Odds Ratio | 95% CI | | p-value |
| **Race** |  |  | | |  |  |  | |  |
| Asian | Reference |  | | |  | Reference |  | |  |
| Black | 1.46 | [1.02, 2.11] | | | **0.041** | 1.74 | [1.10, 2.75] | | **0.018** |
| White | 1.17 | [0.83, 1.65] | | | 0.365 | 1.22 | [0.80, 1.86] | | 0.344 |
| Other | 1.49 | [1.01, 2.20] | | | **0.046** | 1.29 | [0.77, 2.16] | | 0.326 |
| **Ethnicity** |  |  | | |  |  |  | |  |
| Hispanic/Latino/a | 0.74 | [0.54, 1.00] | | | 0.051 | 1.16 | [0.76, 1.77] | | 0.478 |
| Non-Hispanic/Latino/a | Reference |  | | |  | Reference |  | |  |
| **Age** |  |  | | |  |  |  | |  |
| <29 | 0.67 | [0.48, 0.95] | | | **0.026** | 0.70 | [0.44, 1.11] | | 0.132 |
| 30-39 | 1.13 | [0.82, 1.55] | | | 0.449 | 0.82 | [0.54, 1.26] | | 0.369 |
| 40-49 | 1.37 | [1.05, 1.79] | | | **0.021** | 1.26 | [0.84, 1.88] | | 0.261 |
| 50-59 | 0.87 | [0.65, 1.16] | | | 0.341 | 1.03 | [0.68, 1.58] | | 0.878 |
| 60+ | Reference |  | | |  | Reference |  | |  |
| **Time of Arrival** |  |  | | |  |  |  | |  |
| 7A-3P | Reference |  | | |  | Reference |  | |  |
| 3P-11P | 1.02 | [0.84, 1.24] | | | 0.855 | 0.82 | [0.63, 1.06] | | 0.133 |
| 11P-7A | 0.88 | [0.69, 1.12] | | | 0.308 | 0.85 | [0.60, 1.20] | | 0.356 |
| **Insurance Type** |  |  | | |  |  |  | |  |
| Commercial | Reference |  | | |  | Reference |  | |  |
| Medicaid | 1.15 | [0.82, 1.60] | | | 0.429 | 1.35 | [0.89, 2.04] | | 0.161 |
| Medicare | 1.27 | [0.85, 1.90] | | | 0.234 | 1.62 | [0.98, 2.66] | | **0.060** |
| Self-pay | 1.35 | [0.88, 2.07] | | | 0.167 | 1.66 | [0.96, 2.88] | | **0.068** |
| Unknown | 1.29 | [0.87, 1.92] | | | 0.202 | 1.32 | [0.77, 2.26] | | 0.314 |
| **Housing Status** |  |  | | |  |  |  | |  |
| Housed | Reference |  | | |  | Reference |  | |  |
| Unhoused | 1.09 | [0.90, 1.33] | | | 0.371 | 1.20 | [0.89, 1.60] | | 0.227 |
| **Mental Health Disorder*** |  |  | | |  |  |  | |  |
| Absent | Reference |  | | |  | Reference |  | |  |
| Present | 0.74 | [0.58, 0.94] | | | **0.014** | 0.62 | [0.45, 0.86] | | **0.005** |
| **Substance Use Disorder*** |  |  | | |  |  |  | |  |
| Absent | Reference |  | | |  | Reference |  | |  |
| Present | 1.33 | [0.95, 1.85] | | | 0.098 | 0.63 | [0.36, 1.11] | | 0.110 |
| **Arrival Method** |  |  | | |  |  |  | |  |
| Self | Reference |  | | |  | Reference |  | |  |
| Ambulance | 2.54 | [2.07, 3.13] | | | **<.001** | 2.05 | [1.53, 2.74] | | **<.001** |
| Other | 1.36 | [0.99, 1.87] | | | 0.055 | 1.18 | [0.76, 1.85] | | 0.463 |
| **Hospital** |  |  | | |  |  |  | |  |
| SFGH | Reference |  | | |  | Reference |  | |  |
| Parnassus | 0.66 | [0.54, 0.80] | | | **<.001** | 0.57 | [0.43, 0.75] | | **<.001** |
|  |  |  | | |  |  |  | |  |
| **Intercept** | 0.09 | [0.06, 0.15] | | | **<.001** | 0.11 | [0.06, 0.19] | | **<.001** |

*Bolded p values significant at 95% confidence level
